# Chemerin Is a Valuable Biomarker in Patients with HCV Infection and Correlates with Liver Injury

**DOI:** 10.3390/diagnostics10110974

**Published:** 2020-11-19

**Authors:** Georg Peschel, Jonathan Grimm, Karsten Gülow, Martina Müller, Christa Buechler, Kilian Weigand

**Affiliations:** Department of Internal Medicine I, Gastroenterology, Hepatology, Endocrinology, Rheumatology and Infectious Diseases, University Hospital Regensburg, 93053 Regensburg, Germany; georg.peschel@klinik.uni-regensburg.de (G.P.); jonathan.grimm@stud.uni-regensburg.de (J.G.); karsten.guelow@ukr.de (K.G.); martina.mueller-schilling@klinik.uni-regensburg.de (M.M.); kilian.weigand@klinik.uni-regensburg.de (K.W.)

**Keywords:** HCV infection, DAA, body mass index, MELD score, liver fibrosis

## Abstract

Hepatitis C virus (HCV)-induced inflammation contributes to progressive liver disease. The chemoattractant protein chemerin is associated with systemic inflammation. We hypothesized that chemerin is a biomarker that predicts the severity of liver disease in HCV patients. Furthermore, we investigated whether serum chemerin levels change during the course of HCV treatment using direct-acting antivirals (DAAs). Therefore, we measured serum concentration of chemerin in a cohort of 82 HCV-infected patients undergoing DAA treatment. Serum chemerin was positively associated with leukocyte count and negatively with markers of hepatic function and the model of end-stage liver disease (MELD) score. Low circulating chemerin levels significantly correlated with advanced liver fibrosis and cirrhosis as measured by the fibrosis-4 (FIB-4) score, the aminotransferase/platelet (AST/PLT) ratio index (APRI) score and the non-alcoholic fatty liver disease (NAFLD) score. Chemerin did not correlate with viral load or viral genotype. Treatment with DAAs did not improve MELD score and leukocyte count within the observation period, up to three months after the end of DAA treatment. Accordingly, chemerin levels remained unchanged during the treatment period. We conclude that low circulating chemerin is a noninvasive biomarker for hepatic dysfunction and advanced liver fibrosis and cirrhosis in HCV infection.

## 1. Introduction

Chronic hepatitis C virus (HCV) infection causes hepatic inflammation, which triggers progression toward liver fibrosis, cirrhosis, and hepatocellular carcinoma [1]. Liver steatosis is common in these patients and has a higher prevalence in those infected with genotype 3 [2]. The HCV virus reprograms the host lipid metabolism for its own reproduction, and thereby, promotes fatty liver disease. Furthermore, HCV infection induces insulin resistance, which contributes to hepatic fat accumulation [2]. Overweight, obesity, insulin resistance, systemic inflammation, and a disturbed adipokine profile play central roles in the progression of liver diseases [3].

The adipokine chemerin is expressed in white adipose tissues, and adipocyte as well as serum levels are induced in obesity [4]. Serum chemerin has been shown to positively correlate with body mass index (BMI), low-density lipoprotein (LDL), and inflammatory proteins in various studies [5,6,7,8,9,10]. A negative association with high-density lipoprotein (HDL) has also been identified [11]. A meta-analysis of eight studies showed a positive correlation of chemerin with body mass index (BMI), total cholesterol, fasting insulin, and C-reactive protein (CRP) in obesity or metabolic syndrome. However, no significant associations were identified with LDL-cholesterol, HDL-cholesterol, fasting glucose, and hemoglobin A1c (HbA1c) [12].

Chemerin has been shown to be highly expressed in hepatocytes, and chemerin serum levels declined in advanced liver disease [13,14,15]. In patients with liver cirrhosis, negative correlations of chemerin with bilirubin and international normalized ratio (INR) have been identified [14]. The model for end-stage liver disease (MELD) score is used to measure liver disease severity. For calculation of the MELD score, bilirubin, INR, and creatinine values are used [16]. Serum chemerin has been described to negatively correlate with the MELD score in a cohort of patients with mostly alcoholic liver cirrhosis [14].

Serum chemerin was nevertheless induced in patients with HCV in comparison to that in healthy controls [17,18]. Serum chemerin was negatively correlated with albumin in patients with HCV [17]. Positive correlation of serum chemerin with the INR further indicated a direct association of chemerin with liver dysfunction [17].

Over the past few years, direct-acting antivirals (DAAs) have been developed. These agents target HCV-encoded proteins and eliminate the virus within 8 to 16 weeks. Treatment of HCV includes a combination of inhibitors of the RNA-dependent RNA polymerase, the viral protease, and of the nonstructural (NS)5A protein, which contributes to the formation of the replication complex. The side effects of these DAAs are generally mild [19]. These therapies achieve sustained virologic response (SVR) in up to 100% of the patients depending on regimen, genotype, and liver fibrosis [19].

In this study, we evaluated serum chemerin as a potential biomarker in HCV infection. We analyzed laboratory markers of liver function and fibrosis stage in a cohort of 82 patients with chronic hepatitis C. In summary, we herein show that low circulating chemerin levels are predictors of a compromised liver function and progressive liver fibrosis. Of clinical importance, chemerin not only negatively correlated with laboratory values for hepatic dysfunction but also correlated with progressive liver fibrosis and cirrhosis.

## 2. Results

### 2.1. Serum Chemerin Levels in Patients with HCV

**Figure 1:**
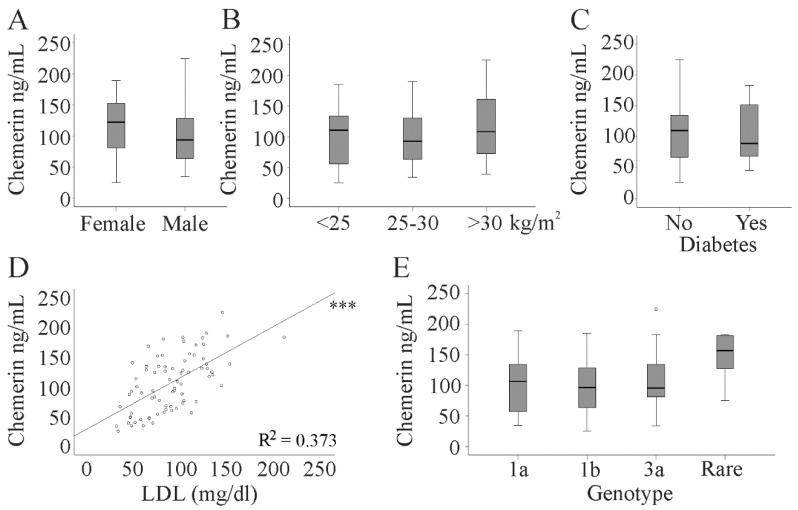
Serum chemerin level in 82 patients with chronic hepatitis C virus (HCV). (**A**) Serum chemerin in 33 female and 49 male HCV patients; (**B**) chemerin levels in patients stratified for body mass index (BMI); (**C**) chemerin in 18 patients with and 64 patients without diabetes; (**D**) correlation of chemerin with low-density lipoprotein (LDL) (R^2^ = 0.373); (**E**) chemerin in patients stratified for HCV genotype (Rare is the group of 6 patients with genotypes other than 1a, 1b, and 3a). The small circle above the box marks an outlier. *** *p* < 0.001.

Chemerin serum levels were determined in 82 chronic HCV patients. Serum chemerin was similar in both sexes and did not increase in parallel with BMI (Figure 1A,B). Accordingly, chemerin did not correlate with BMI (Table 1). The 18 patients with diabetes in our cohort had serum chemerin levels similar to the non-diabetic patients (Figure 1C). Serum chemerin positively correlated with LDL but not with HDL (Figure 1D and Table 1).

In several cohorts, a positive correlation of serum chemerin and markers of inflammation has been described [7,8,10,16]. In the HCV patients, chemerin was positively associated with leukocyte count but did not correlate with CRP (Table 1). HCV genotypes were 1a in 24 patients, 1b in 38 patients, and 3a in 14 patients. Other genotypes were rare in this cohort, and these 6 patients were assigned to one group.

Serum chemerin was similar irrespective of viral genotype (Figure 1E) and displayed no correlation with viral load (r = 0.194, *p* = 0.081).

### 2.2. Association of Chemerin with the MELD Score

The MELD score is a predictor of survival in patients with liver cirrhosis and is calculated using bilirubin, creatinine, and the INR as a measure of blood coagulation [16].

**Figure 2:**
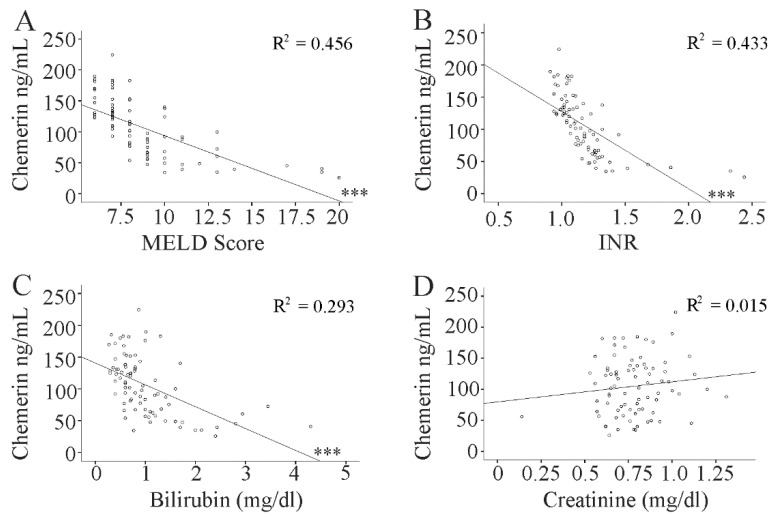
Correlation of serum chemerin with markers of liver injury. Correlation of chemerin with (**A**) the MELD score; (**B**) INR; (**C**) bilirubin; and (**D**) creatinine. *** *p* < 0.001.

Serum chemerin negatively correlated with the MELD score. This association was still significant after correction for the confounding factors gender, age, BMI, viral load, and aminotransferase levels (r = −0.658, *p* < 0.001). In addition, a negative correlation existed with bilirubin and INR but not with creatinine (Figure 2A–D, Table 1).

### 2.3. Serum Chemerin in Relation to Hepatic Steatosis and Liver Fibrosis

In the HCV cohort, 37 patients had a diagnosis of fatty liver disease by ultrasound imaging. The prevalence of liver steatosis was 46% in genotype 1a, 47% in genotype 1b, 36% in genotype 3a, and 50% in the patients with rare genotypes, and did not vary between the four groups.

**Figure 3:**
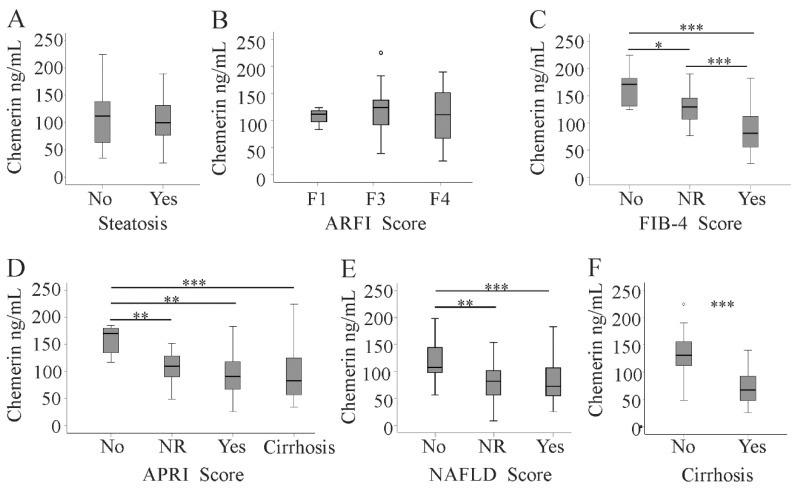
Serum chemerin in relation to hepatic steatosis and fibrosis scores. (**A**) Chemerin in 37 patients with and 45 patients without liver steatosis; chemerin levels in patients stratified for fibrosis by (**B**) the acoustic radiation force impulse (ARFI) score (**C**), the fibrosis-4 (FIB-4) score, (**D**) the aminotransferase/platelet (AST/PLT) ratio index (APRI) score, and (**E**) the non-alcoholic fatty liver disease (NAFLD) score; (**F**) chemerin in 37 patients with liver cirrhosis and 45 non-cirrhotic patients diagnosed by ultrasound (no fibrosis = no; not reliable values = NR, fibrosis = yes). Small circles above the boxes mark outliers. * *p* < 0.05, ** *p* < 0.01, *** *p* < 0.001.

Serum chemerin was similar in patients with and without liver steatosis (Figure 3A). Serum chemerin levels positively correlated with albumin. Furthermore, a modest negative association with AST levels was identified (Table 1).

We used the following noninvasive methods to evaluate liver fibrosis: acoustic radiation force impulse (ARFI), the aspartate aminotransferase/platelet (AST/PLT) ratio index (APRI), the NAFLD score (which uses age, BMI, diabetes, AST/ALT ratio, platelet count, and albumin [20]), and the fibrosis 4 (FIB-4) score (calculated from age, AST, ALT, and platelet count [21,22]). ARFI indicated liver fibrosis in 67, the FIB-4 score in 53, the NAFLD score in 35, and the APRI score in 57 patients. Serum chemerin did not change with increasing ARFI fibrosis score (Figure 3B). Upon diagnosis of fibrosis by the FIB-4 score, the APRI, and the NAFLD score, chemerin declined in patients with more advanced liver fibrosis (Figure 3C–E). Accordingly, serum chemerin was low in the 37 patients with liver cirrhosis as diagnosed by ultrasound examination (Figure 3F).

The association between liver cirrhosis and serum chemerin levels was determined using logistic regression analysis (Table 2). This relation remained highly significant after adjustment for age, gender, BMI, and viral load, which are risk factors for fibrosis progression [23,24]. When alanine aminotransferase and aspartate aminotransferase were included, serum chemerin still discriminated liver cirrhosis patients from non-cirrhotic ones. Significance was lost after further adjustment for the MELD score (Table 2).

### 2.4. Effect of Direct-Acting Antivirals (DAAs) on Serum Chemerin during Therapy and up to 3 Months after the End of DAA Therapy

**Figure 4:**
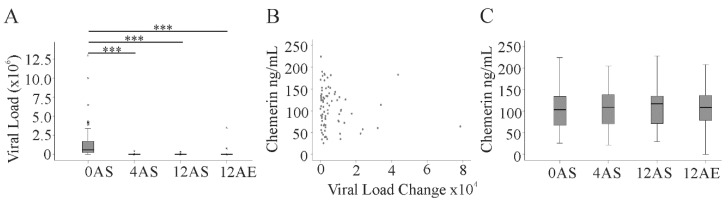
Viral load and serum chemerin during DAA therapy. (**A**) Viral load before (0), 4 and 12 weeks after start of therapy (AS), and 12 weeks after end of therapy (AE); (**B**) correlation of chemerin at the start of DAA therapy and change of viral load within 4 weeks of treatment; (**C**) chemerin at the times described in (A). Small circles or asterisks above the boxes mark outliers. *** *p* < 0.001.

DAA therapy efficiently eliminated HCV in our treatment cohort with different anti-HCV treatment regimens [19]. DAA therapy for 4 weeks effectively diminished viral load, which was still undetectable at 12 weeks and three months after the end of treatment (Figure 4A). Serum chemerin level did not correlate with a change in viral load within the first 4 weeks of DAA therapy (Figure 4B). Serum chemerin was measured 4 weeks (79 patients) and 12 weeks (81 patients) after the start of DAA treatment and 3 months after the end of therapy (71 patients). Serum chemerin did not decline during therapy and was also not changed 3 months after treatment (Figure 4C). ALT and AST decreased during therapy. This was significant for ALT at 4 and 12 weeks and 3 months after the end of DAA therapy and for AST at 12 weeks after treatment (Table 3). Albumin was found increased 3 months after the end of the therapy. Accordingly, the FIB-4 score (*p* = 0.001) and the ARFI score (*p* < 0.001) were reduced 3 months after the end of therapy. The APRI score was also lower at 3 months after the end of therapy (*p* < 0.001), whereas the NAFLD score was not changed (*p* = 0.828). LDL was already induced after 4 weeks treatment and remained high at the two later time points. Bilirubin, creatinine, leukocyte count, CRP, and HDL did not alter (Table 3).

Chemerin correlated with the MELD score at 4 and 12 weeks after the start of the DAA therapy, and with the MELD score 3 months after the end of treatment. For all time points, negative correlations with AST, INR, and bilirubin and positive correlations with leukocyte count and LDL could be detected (Table 1). The positive association with albumin 4 and 12 weeks after the start of DAA therapy disappeared at 3 months after the end of treatment (Table 1).

The association between liver cirrhosis and serum chemerin levels 3 months after the end of therapy was determined using logistic regression analysis (Table 4). This relation remained significant after adjustment for age, gender, BMI, alanine aminotransferase, and aspartate aminotransferase. Significance disappeared after adjustment for the MELD score (Table 4).

## 3. Discussion

Here, we identified negative correlations of serum chemerin with laboratory measures of liver function in patients with chronic HCV infections. Improvements in bilirubin, albumin, and MELD score after virus eradication are minimal [25], and accordingly, chemerin was not reduced after treatment with DAA therapy. Thus, chemerin is a biomarker for HCV-induced liver damage before and after DAA therapy.

In Egyptian HCV patients, serum chemerin increased in parallel with histological scores of liver fibrosis [26]. A separate study described a negative correlation between chemerin and albumin, indicating a rise of chemerin in patients with decreased liver function [17]. Moreover, chemerin was positively correlated with INR [17]. Kukla et al. identified a decline of serum chemerin in HCV patients with higher grade of hepatic inflammation. An association with histologically defined fibrosis stages did not exist [18]. The mean APRI score of the cohort enrolled by Kukla et al. was 0.67 [18] and was 1.9 in the present cohort. Thus, a decline of chemerin with increasing fibrosis stage may only occur in patients with more advanced liver disease.

In patients with hepatocellular carcinoma or hepatic metastasis from colorectal cancer, histologically defined stages of liver fibrosis were not associated with serum chemerin [27]. Here, chemerin was negatively correlated with bilirubin [27]. Negative associations of chemerin with bilirubin were also identified in patients with decompensated liver cirrhosis with mostly alcoholic disease etiology [14]. In a separate cohort of cirrhosis patients with mainly alcoholic disease etiology, chemerin was reduced in those with decompensated liver cirrhosis. Correlations with bilirubin, albumin, and the MELD score were not identified [13]. Notably, the MELD score was 9 (6–21) in these patients and 8 (6–20) in the HCV patients studied herein. These previous analyses of chemerin in cirrhosis enrolled 20 patients [17], 70 patients [26], 45 patients [13], 40 patients [18], 68 patients [27], and 80 patients [14], and in the present analysis 82 patients were enclosed. Sample size has an impact on the significance of correlations, and significant associations may not exist in smaller study groups.

So far, there is no explanation why the different studies described above did not consistently identify a decline of chemerin in patients with advanced liver fibrosis. Moreover, two studies even described an increase of chemerin in patients with advanced disease [17,26]. Liver biopsy is still considered the gold standard in the diagnosis of liver fibrosis [22]. Liver histology was not done in the current study and this is a limitation of our investigations. However, Kukla et al. could not identify a correlation of serum chemerin with histologically defined liver fibrosis stages [18]. ARFI imaging evaluates liver stiffness. Serum chemerin did not decline with increasing fibrosis stage evaluated by ARFI or histology [18]. Serum chemerin only declined in advanced liver disease when the fibrosis scores were calculated from laboratory values.

The ARFI, APRI, NAFLD, and FIB-4 scores are accurate approaches in diagnosing significant and advanced fibrosis [20,22]. The FIB-4 score and the APRI score were positively correlated with histological scoring in HCV patients, whereas such a correlation did not exist with the MELD score [21]. In the general population, there was a modest association between liver stiffness as measured by transient elastography and the APRI fibrosis score. Associations with the ALT/AST ratio and the FIB-4 score did not exist [28]. Moreover, fibrosis scoring by liver elastography in patients with HCV was influenced by serum transaminase levels [29]. Measurement of spleen stiffness by point shear-wave elastography was also affected by serum transaminase levels in patients with HCV infection [30]. These studies showed that serum transaminase levels, which are related to inflammation and virus-induced cytolysis, are confounding factors in noninvasive techniques to measure tissue stiffness [29,30]. The decline of the ARFI score three months after the end of DAA therapy is most likely a consequence of lower serum transaminase levels rather than an improvement in liver stiffness. Serum chemerin did not change during DAA therapy and may become a noninvasive biomarker of residual liver function before and after DAA treatment. Whether serum chemerin may also differentiate fibrosis stages needs further analysis.

Chemerin serum levels are related to inflammation [7,8,10], and positive correlations with leukocyte counts were identified in the present cohort. Varying grades of inflammation between the different patient cohorts may contribute to the inconsistent results reported in different studies [17,18,26].

Chronic inflammation is a characteristic of HCV and was partly normalized after HCV eradication [31]. Various cytokines and chemokines were high in HCV-infected patients and declined after therapy [31]. A 12 week treatment with a combination of sofosbuvir/ledipasvir and ribavirin suppressed plasma chemerin in a study with 12 patients [32].

Such a decline of serum chemerin did not occur in the current cohort. Chemerin was measured after 4 and 12 weeks of therapy and 12 weeks after the end of therapy. Leukocyte count and CRP were mostly in the normal range and did not change with DAA treatment.

DAAs are highly efficient and relatively safe drugs in the therapy of HCV-infected patients [33]. One adverse effect is the rise of LDL-cholesterol. Increase in LDL-cholesterol was higher in ledipasvir/sofosbuvir-treated patients than in those treated with daclatasvir/asunaprevir [34]. LDL was also induced in the current study cohort after therapy, and this was observed in all treatment groups. Serum chemerin correlated with LDL before and at all times after start of DAA treatment, and positive associations of chemerin and LDL are in agreement with previous analysis in non-HCV patients [8,11].

Chemerin is an adipokine and circulating levels are increased in obesity [6,7,10,35,36]. Such an association with obesity was not observed in our HCV cohort spanning a wide BMI range (18.4–40.4). Similarly, Kukla et al. did not identify an association of chemerin with BMI in a cohort of 40 patients with HCV [18].

In 157 genotype 4 patients treated with sofosbuvir, pegylated interferon-alpha-2a plus ribavirin high serum chemerin predicted treatment failure [37]. Here, an association of basal chemerin levels with the change in viral load after 4 weeks of DAA therapy was not identified. Chemerin was, moreover, not correlated with viral load before therapy start. The patients not responding to therapy in the study by Marwa and Al-Aliaa had about 10-fold higher chemerin levels than the responding patients and the patients enrolled in the present study [37]. Serum chemerin may thus predict treatment failure but is neither associated with viral load before DAA therapy nor with change of viral load after 4 weeks of treatment.

HCV genotypes 1 and 3 are very common [38], and most patients in the present cohort were infected with virus genotypes 1a, 1b, and 3a. Serum chemerin did not vary between patients with different genotypes. Liver steatosis is present in about 50% of HCV-infected patients and has a higher prevalence of about 73% in those infected with genotype 3 [2,23]. In our study cohort, 45% of the patients had fatty liver, and prevalence was not related to the viral genotype. Kukla et al. described similar chemerin levels in HCV-infected patients with and without liver steatosis [18], and serum chemerin did not differ between these two groups in our cohort.

## 4. Materials and Methods

### 4.1. Study Cohort

This study was performed at the Department of Internal Medicine I at the University Hospital of Regensburg. The study was approved by the local ethical committee of the University Hospital of Regensburg (14-101-0049) and was performed according to the updated guidelines of good clinical practice and updated Declaration of Helsinki. Serum chemerin was measured by ELISA in 82 patients with chronic HCV infection (Table 3). The median age of the patients was 59 (24–80) years. Patients were treated from October 2014 to September 2019. All patients had chronic hepatitis C and were treated with one of the following regimens: sofosbuvir/daclatasvir, sofosbuvir/ledipasvir, sofosbuvir/velpatasvir, glecaprevir/pibrentasvir, or elbasvir/grazoprevir. Treatment was performed according to international guidelines [19]. Patient characteristics are given in Table 3. All patients gave informed consent prior to inclusion in the study.

### 4.2. ELISA

ELISA to measure human chemerin was from R&D Systems and was performed as recommended by the distributor. Serum was diluted 1:250-fold for analysis.

### 4.3. Statistical Analysis

Data are presented as boxplots. Small circles or asterisks above the boxes mark outliers. Statistical differences were analyzed by one-way ANOVA with post hoc Bonferroni, Mann–Whitney U-test, Spearman correlation, two-tailed partial correlation, and logistic regression (SPSS Statistics 25.0 program, IBM, Leibniz-Rechenzentrum, München, Germany) or chi-square test, and a value of *p* < 0.05 was regarded significant.

## 5. Conclusions

The current study identified serum chemerin as a valuable biomarker of liver disease severity in patients with HCV before and after DAA therapy.

## Figures and Tables

**Table 1:** Spearman correlation coefficients and p-values of the correlations of BMI and laboratory parameters and chemerin in the whole cohort before direct-acting antiviral (DAA) therapy, at 4 and 12 weeks after start of therapy and 3 months after the end of therapy. Significant correlations are in bold.

Parameter	Baseline (82 patients)	4 weeks (79 patients)	12 weeks (81 patients)	3 Months after End of Therapy (71 patients)
BMI kg/m^2^	0.046 (0.695)	0.028 (0.816)	0.054 (0.653)	−0.002 (0.987)
MELD Score	**−0.777 (<0.001)**	**−0.749 (<0.001)**	**−0.752 (<0.001)**	**−0.578 (<0.001)**
ALT U/L	0.119 (0.286)	−0.067 (0.559)	−0.057 (0.612)	0.198 (0.104)
AST U/L	**−0.233 (0.035)**	**−0.379 (0.001)**	**−0.500 (<0.001)**	**−0.475 (<0.002)**
Bilirubin mg/dL	**−0.588 (<0.001)**	**−0.614 (<0.001)**	**−0.668 (<0.001)**	**−0.591 (<0.001)**
Albumin g/L	**0.592 (<0.001)**	**0.453 (<0.001)**	**0.537 (<0.001)**	0.220 (0.071)
INR	**−0.815 (<0.001)**	**−0.744 (<0.001)**	**−0.786 (<0.001)**	**−0.815 (<0.001)**
Creatinine mg/dL	0.099 (0.378)	0.000 (0.997)	0.111 (0.325)	0.165 (0.175)
Leukocytes n/L	**0.628 (<0.001)**	**0.515 (<0.001)**	**0.572 (<0.001)**	**0.360 (0.003)**
CRP mg/L	0.003 (0.979)	0.079 (0.489)	−0.103 (0.362)	0.199 (0.101)
HDL mg/dL	0.021 (0.857)	0.123 (0.292)	0.129 (0.261)	0.005 (0.971)
LDL mg/dL	**0.598 (<0.001)**	**0.655 (<0.001)**	**0.681 (<0.001)**	**0.618 (<0.001)**

Alanine amino transferase, ALT; aspartate aminotransferase, AST; C-reactive protein, CRP; MELD, model of end-stage liver disease; INR, international normalized ratio; LDL, low-density lipoprotein; HDL, high-density lipoprotein.

**Table 2:** Odds ratio (OR) and 95% confidence interval (CI) of serum chemerin with liver cirrhosis. Model 1: adjusted for age and BMI; Model 2: adjusted for age, BMI, and gender; Model 3: adjusted for age, BMI, gender, and viral load; Model 4: adjusted for age, BMI, gender, viral load, alanine aminotransferase, and aspartate aminotransferase; Model 5: adjusted for age, BMI, gender, viral load, alanine aminotransferase, aspartate aminotransferase, and the MELD score.

Model	OR	95% CI	*p*
Model 1	0.938	0.912–0.966	<0.001
Model 2	0.937	0.910–0.964	<0.001
Model 3	0.936	0.908–0.965	<0.001
Model 4	0.935	0.907–0.965	<0.001
Model 5	0.967	0.934–1.001	0.058

**Table 3:** Laboratory parameters of the patients at baseline, after 4 and 12 weeks of antiviral therapy, and 3 months after the end of treatment. Superscript numbers indicate the number of patients in case the laboratory values were not documented for the whole study group (alanine amino transferase (ALT), aspartate aminotransferase (AST), C-reactive protein (CRP). * *p* < 0.05, *** *p* < 0.001 (baseline compared to 3 months after therapy), ^%%^ *p* < 0.01, ^%%%^ *p* < 0.001 (baseline compared to 4 weeks therapy), ^$^ *p* < 0.05, ^§§^ *p* < 0.01 (baseline compared to 12 weeks therapy).

Laboratory Parameter	Baseline (82 patients)	4 weeks Therapy (79 patients)	12 weeks Therapy (81 patients)	3 Months after End of Therapy (71 patients)
Ferritin ng/mL	141 (7.0–2309) ^76,^**	122 (9.1–1638)	97 (6.6–1161) ^80^	69.8 (6.4–1133) ^70,^**
ALT U/L	86 (22–287) ***^,%%%,$$$^	28 (7–255) ^%%%^	27 (8–388) ^$$$^	26 (6–443) ^***^
AST U/L	75 (17–1230) ^$^	28 (10–1140)	26 (7–836) ^$^	25 (11–1390)
Bilirubin mg/dL	0.8 (0.3–4.3)	0.8 (0.06–4.85)	0.6 (0.05–7.5)	0.6 (0.04–2.8) ^71^
Albumin g/L	36 (19–45) ^81,^***	37 (19–47)	37 (16–45) ^79^	38.6 (26.1–46) ^71,^***
INR	1.13 (0.91–2.44)	1.12 (0.90–3.15)	1.13 (0.87–2.22)	1.08 (0.88–2.81)
Creatinine mg/dL	0.78 (0.14–1.31) ^81^	0.81 (0.21–1.54)	0.77 (0.14–1.92)	0.8 (0.1–1.4)
Leukocytes n/L	5.9 (2.2–72.4)	6.1 (2.0–72.5)	5.9 (2.4–62.9)	6.0 (1.9–38.6) ^71^
CRP mg/L	2.9 (2.9–29.9)	2.9 (0–19.8)	2.9 (2.9–19.1) ^80^	2.9 (2.9–46.3)
HDL mg/dL	51 (19–103) ^77^	54 (25–102) ^77^	51 (13–96) ^78^	52 (23–86) ^65^
LDL mg/dL	88 (31–210) ^77,^*^,%%,§§^	113 (33–206) ^77,%%^	106 (33–198) ^78,§§^	115 (38–185) ^64,^*

**Table 4:** Odds ratio (OR) and 95% confidence interval (CI) of serum chemerin with liver cirrhosis at 3 months after therapy. Model 1: adjusted for age and BMI; Model 2: adjusted for age, BMI, and gender; Model 3: adjusted for age, BMI, gender, alanine aminotransferase, and aspartate aminotransferase; Model 4: adjusted for age, BMI, gender, alanine aminotransferase, aspartate aminotransferase, and MELD score.

Model	OR	95% CI	*p*
Model 1	0.966	0.947–0.985	0.001
Model 2	0.966	0.947–0.985	0.001
Model 3	0.963	0.943–0.984	0.001
Model 4	0.969	0.935–1.004	0.082
